# Quality of Life of Women After a First Diagnosis of Breast Cancer Using a Self-Management Support mHealth App in Taiwan: Randomized Controlled Trial

**DOI:** 10.2196/17084

**Published:** 2020-03-04

**Authors:** I-Ching Hou, Hsin-Yi Lin, Shan-Hsiang Shen, King-Jen Chang, Hao-Chih Tai, Ay-Jen Tsai, Patricia C Dykes

**Affiliations:** 1 School of Nursing National Yang-Ming University Taipei City Taiwan; 2 Department of Nursing Hsinchu Mackay Memorial Hospital Hsinchu Taiwan; 3 Department of Computer Science and Information Engineering National Taiwan University of Science and Technology Taipei Taiwan; 4 Taiwan Breast Cancer Foundation Taipei Taiwan; 5 Department of Surgery National Taiwan University Taipei Taiwan; 6 Center for Patient Safety Research and Practice Brigham and Women’s Hospital Boston, MA United States; 7 Harvard Medical School Boston, MA United States

**Keywords:** breast cancer, mHealth app, self-management, quality of life

## Abstract

**Background:**

There are over 2 million newly diagnosed patients with breast cancer worldwide with more than 10,000 cases in Taiwan each year. During 2017-2018, the National Yang-Ming University, the Taiwan University of Science and Technology, and the Taiwan Breast Cancer Prevention Foundation collaborated to develop a breast cancer self-management support (BCSMS) mHealth app for Taiwanese women with breast cancer.

**Objective:**

The aim of this study was to investigate the quality of life (QoL) of women with breast cancer in Taiwan after using the BCSMS app.

**Methods:**

After receiving a first diagnosis of breast cancer, women with stage 0 to III breast cancer, who were recruited from social networking sites or referred by their oncologists or oncology case managers, were randomized 1:1 into intervention and control groups. Intervention group subjects used the BCSMS app and the control group subjects received usual care. Two questionnaires—the European Organization for Research and Treatment of Cancer (EORTC) Quality-of-Life Questionnaire Core 30 (QLQ-C30) and the EORTC Breast Cancer-Specific Quality-of-Life Questionnaire (QLQ-BR23)—were distributed to subjects in both arms. Paper-based questionnaires were used at baseline; paper-based or Web-based questionnaires were used at 1.5-month and 3-month follow-up evaluations. All evaluations were self-assessed and anonymous, and participants were blinded to their allocation groups. Descriptive analysis, the Pearson chi-square test, analysis of variance, and the generalized estimating equation were used to analyze the data. Missing values, with and without multi-imputation techniques, were used for sensitivity analysis.

**Results:**

A total of 112 women were enrolled and randomly allocated to either the experimental group (n=53) or control group (n=59). The follow-up completion rate was 89.3% (100/112). The demographic data showed homogeneity between the two groups in age (range 50-64 years), breast cancer stage (stage II), marital status (married), working status (employed), and treatment status (receiving treatments). The mean total QoL summary scores from the QLQ-C30 (83.45 vs 82.23, *P*=.03) and the QLQ-BR23 (65.53 vs 63.13, *P*=.04) were significantly higher among the experimental group versus the control group, respectively, at 3 months.

**Conclusions:**

This research provides support for using a mobile health care app to promote the QoL among women in Taiwan after a first diagnosis of breast cancer. The BCSMS app could be used to support disease self-management, and further evaluation of whether QoL is sustained is warranted.

**Trial Registration:**

ClinicalTrials.gov NCT004174248; https://clinicaltrials.gov/ct2/show/NCT04174248

## Introduction

### Background

Breast cancer has been the most common type of malignant cancer in women in Taiwan for more than 10 years and is the fourth-leading cause of death [1]. Each year, more than 10,000 women are diagnosed with breast cancer, and about 2000 women die from breast cancer: in 2014, there were 12,714 new diagnoses and 2083 deaths. The incidence of breast cancer in Taiwan is common in younger women, 40-65 years of age, with stages 0 to II being the most common at diagnosis [2]. With early detection and treatment of breast cancer, the treatment effect is good. With appropriate treatment, the 5-year relative survival rate approaches 87.2% and breast cancer is often regarded as a chronic disease. Breast cancer treatment includes breast preservation or resection, chemotherapy, hormone therapy, and targeted therapy [3-5]. The most common side effects differ by treatment method and include surgical-wound pain, nausea, and vomiting (see Table 1). Treatment side effects can cause anxiety and depression, which can affect a woman’s willingness to undergo treatment and can severely decrease the quality of life (QoL) for women with breast cancer [6-12].

Today, the *self-management* model is generally valued and promoted in Taiwan and abroad [13-15]. Self-management is different from traditional disease management; this includes education, emphasizing patient-centered and disease-oriented self-management, and taking the initiative to participate in health care activities. With self-management, the patient learns problem solving, disease control, life adjustment, physical and mental symptom management, and lifestyle changes in order to coexist with a chronic disease in daily life. A systematic review on chronic disease intervention studies showed that self-management can significantly improve knowledge of chronic disease, enhance self-care behavior, and increase self-efficacy [16]. In a study of chronic disease self-management applied to women with breast cancer, it was found that self-management measures can effectively improve the QoL for women living with breast cancer, increase self-efficacy, and help women to better manage their medical and emotional tasks [17].

The World Health Organization defines QoL as “an individual's perception of their position in life in the context of the culture and value systems in which they live in relation to their goals, expectations, standards and concerns. It is a multifaceted concept affected by the person's physical health, psychological state, personal beliefs, social relationships and their relationship to salient features of their environment” [18]. QoL can be objectively measured. While breast cancer is the most common cancer among Taiwanese women, the survival rate is high. Providing patients with adequate physical and psychological treatment and care could improve their QoL, which becomes an important indicator of the quality of medical care [19-21]. Therefore, the measurement of QoL can objectively reflect the response of women with breast cancer to disease and provide important evaluation indicators for the effectiveness of interventions.

The use of mobile apps by patients with cancer is becoming more common. More than half of patients with cancer are willing to transmit information through an app to support their treatment, and 84% of medical professionals, mainly physicians, support the use of apps among this population [22,23]. One study suggests that apps can support outpatient visits, documentation of adverse events, treatment, and medication reminders [22]. Mobile apps have also been developed for breast cancer self-management [24-27]. However, we are unaware of any mobile self-management apps developed specifically for Taiwanese women with breast cancer. Therefore, our team developed the breast cancer self-management support (BCSMS) mHealth app to address this gap. The development and usability testing of the BCSMS app has been reported previously (publication is forthcoming). The purpose of this study was to evaluate the impact of using the BCSMS app on the QoL of Taiwanese women after an initial diagnosis of breast cancer.

**Table 1:** Common side effects of breast cancer treatments, which differ by treatment method.

Treatment	Common side effects
Surgery	Surgical-wound pain and lymphedema
Chemotherapy	Nausea, vomiting, loss of appetite, oral mucositis, fatigue, hair loss, myelosuppression, neuropathy cognitive disorders, weight gain, and poor memory
Radiotherapy	Dermatitis, sleep disturbance, and fatigue
Hormone therapy	Menopause, lack of libido, hot flashes, headache, night sweats, and insomnia

### Prior Work

In Taiwan, since June 2017, the National Yang-Ming University, the Taiwan University of Science and Technology, and the Taiwan Breast Cancer Prevention and Research Foundation collaborated to develop the BCSMS app. Eight main features of the app included the following: (1) the evidence or *knowledge* about breast cancer, (2) *exercise and rehabilitation* after surgery, (3) *diet and nutrition* for breast cancer patients, (4) *emotional support* to prevent anxiety and depression, (5) a *personal health record* for tracking treatment and side effects, (6) *social resource* information, (7) *experience sharing*, and (8) *expert consulting*. We pilot-tested the BCSMS app with 45 Taiwanese women with breast cancer in 2018 using the modified *technology acceptance model of mobile services* survey. From this pilot test, our team found that the BCSMS app had sound usability and was accepted by the participants. The BCSMS app has since been outsourced to a technology company for long-term maintenance and can be downloaded for widespread use. To access the app, the keyword “ibreast” should be used to search for the iOS version and the keywords “pink passport” should be used for the Android version.

## Methods

### Study Design and Setting

The study was a single-blinded, parallel-group, randomized controlled trial with a pretest evaluation (T0), as well as 1.5-month (T1) and 3-month (T2) follow-up evaluations. The study sites included two medical centers— National Taiwan University Hospital and MacKay Memorial Hospital—and one area hospital—Hsinchu Mackay Memorial Hospital—in northern Taiwan. Every patient at each of the study sites received similar care from their health care team (eg, oncology clinic follow-up every 3 weeks during chemotherapy; oncology nurse case managers available to work face-to-face or via telephone with patients when needed on their cancer journey; and transfers by their physicians for consultations with health care professionals in various disciplines, such as pharmacists, physical therapists, nutritionists, psychologists, and social workers, when they had related health problems). The health care professionals’ care models influencing QoL improvement were assumed to be similar in this study.

### Ethical Considerations

This study followed the ethical principles of the Declaration of Helsinki [28] and was approved by the Institutional Review Board (IRB) of National Yang Ming University in Taiwan (IRB No. YM107109E). Participation in the study was voluntary. Eligible participants were provided with two different informed consent forms according to their assigned groups. The consent forms included the same information about the protocol regarding their QoL data collection, and every participant was provided US $3 for each pretest evaluation and follow-up evaluation after the study. The information items that differed on the experimental group’s consent form were (1) a brief description about the contents of the BCSMS app and (2) a statement communicating that the BCSMS app would be installed on participants’ mobile phones with remote technology support. The protocol was determined to be of minimal risk to the participants. Nevertheless, all participant data were anonymized and stored on an encrypted, password-protected server.

### Recruiting the Study Participants, Sample Size, and Randomization

Women with breast cancer who met the following inclusion criteria were recruited: (1) first diagnosis of stage 0 to III breast cancer within the past year, (2) aged 20-65 years, to avoid barriers with respect to aging influencing mobile health usability for older adults [29], (3) had an Android or iOS mobile phone, (4) able to read and write in Chinese, and (5) willing to participate in the study and provide informed consent.

A priori power analysis was calculated using G*Power, version 3.1 (Heinrich-Heine-Universität Düsseldorf), by performing F tests and repeated-measures, between-subjects factor analysis of variance; a medium effect size of 0.25, a significance level of .05, and a power of 0.8 were used for sample size calculation according to Cohen [30]. The a priori sample size was 41 in each group. We oversampled our eligible patient population to account for potential dropouts and expected to include a total of 106 patients in this study.

Participants were recruited in two different ways. First, a recruitment ad was posted on the Taiwan Cancer Foundation social networking sites (eg, Facebook and Line) and interested participants contacted us through the online registration. Second, patients were referred by their oncologists and oncology case managers from the study settings. To blind the study group participants to allocation [31] and prevent selection bias (eg, technology novelty bias) [32], the ad stated that participant recruitment was for a QoL evaluation following cancer treatment only, with no mention of the BCSMS app. The recruitment poster was used by recruiters to introduce the patients to the purpose of the study. For homogeneity of patients at each stage of breast cancer—stage 0 to III—between the two groups, the expected numbers of patients with stage 0, stage I, stage II, and stage III breast cancer were about 20, 36, 36, and 14, respectively: the incidence rate of each stage [1] was used and multiplied by 106. Next, participants at each stage were randomly assigned 1:1 into one of two study groups: control or experimental. The randomization scheme was generated using the website Randomization.com [33] for participants at each stage.

### Data Collection Procedures

The study team collected data from January to July 2019. Patients were blinded to their allocation groups—experimental or control—and their pretest data were collected via a paper-based instrument. After the pretest data were collected, the BCSMS app was installed on the mobile phones of the participants in the experimental group and they were taught how to use the app. Each participant in the experimental group could use BCSMS app any time as needed; there were no prompts or reminders from the study team. The entire pretest study design was done via face-to-face contact at the study sites. Data collection for the two follow-up evaluations was completed via a Web-based instrument, together with a phone call, email, and/or communication software (eg, Line). Paper-based instruments were also provided for participants that chose not to use the Web-based instrument. All evaluations were anonymous. During data collection, the content of the BCSMS app was frozen, however, the intervention group was supported in overcoming any technical problems associated with the BCSMS app (eg, log-in problems and data entry problems). During the study, participants in both groups received the same care, concurrently, from health care professionals at the study sites.

### Instruments

The QoL instrument consisted of two parts. The first part included demographic items (eg, age, disease stage, marital status, and working status) and treatment-related items. The second part was the Taiwan Chinese version of two Quality-of-Life Questionnaires (QLQs) originally developed by the European Organization for Research and Treatment of Cancer (EORTC) [34,35]: the EORTC QLQ Core 30 (QLQ-C30), version 3, and the EORTC Breast Cancer-Specific QLQ (QLQ-BR23). These instruments have good test-retest reliability, high internal consistency in most scales, and show expected differences between patients in active chemotherapy and those in follow-up groups [36]. The QLQ-C30 incorporates five functional scales—physical functioning, role functioning, emotional functioning, cognitive functioning, and social functioning—nine symptom scales—fatigue, nausea and vomiting, pain, dyspnea, insomnia, appetite loss, constipation, diarrhea, and financial difficulties—as well as global health status and QoL scales [34]. The QLQ-BR23 is the module for breast cancer. It incorporates four functional scales—body image, sexual functioning, sexual enjoyment, and future perspective— and four symptom scales—systemic therapy side effects, breast symptoms, arm symptoms, and upset by hair loss [37]. According to the EORTC QLQ scoring manual, a high score for a functional scale represents a high or healthy level of functioning when using the QLQ-C30 and QLQ-BR23, a high score for global health status or QoL represents a high QoL when using the QLQ-C30, but a high score for a symptom scale or item represents a high level of symptomatology or problems when using the QLQ-C30 and QLQ-BR23 [38]. In this study, the instrument used was a paper-based or Web-based form. Participants could choose one of the evaluation form types according to their preferences (eg, desire to go paperless or efficiency when self-reporting [39]) at the two follow-up evaluations. The average time required to complete any of the evaluation forms was approximately 15 minutes, and most patients required no assistance. Following data collection, the internal consistency reliability of both instrument types used by the two groups was assessed: the Cronbach alpha levels at baseline, 1.5-month follow-up, and 3-month follow-up were .90-.96 using the QLQ-C30 and .71-.95 using the QLQ-BR23. The results indicated that both types of forms—paper based and Web based—had adequate reliability [40].

### Data Analysis

All completed questionnaires were coded and analyzed using SPSS Statistics for Windows, version 20.0 (IBM Corp). Each item of the QLQ-C30 and QLQ-BR23 underwent linear transformation to obtain a score of 0-100 in accordance with the EORTC QLQ scoring manual [38]. For data values that were missing at random (MAR), the multi-imputation technique was used [38,41] and the average score of the observed data at the same follow-up interval in the same group was adopted to impute the MAR. The dummy variables—summary scores of the QLQ-C30 and QLQ-BR23—were adopted to represent the overall QoL outcome of each respondent and the formula was the sum of scores from each functional item scale and each revised symptom item (eg, the difference between 100 and each symptom item scale score). A higher summary score represents a higher QoL. Frequency and percentage as well as mean and standard deviation were used for descriptive statistics for clinical variables, the QLQ-C30, and the QLQ-BR23. We compared the baseline results of the control and experimental groups using chi-square tests for categorical variables and *t* tests for continuous variables. All analyses were intention-to-treat using a repeated-measures analysis and the generalized estimating equation (GEE) [42]. This method was used to account for the lack of adherence values over time and to detect any time × group effects among the target indicators. Significance was defined as a *P* value less than .05.

## Results

### Randomization and Attrition

**Figure 1:**
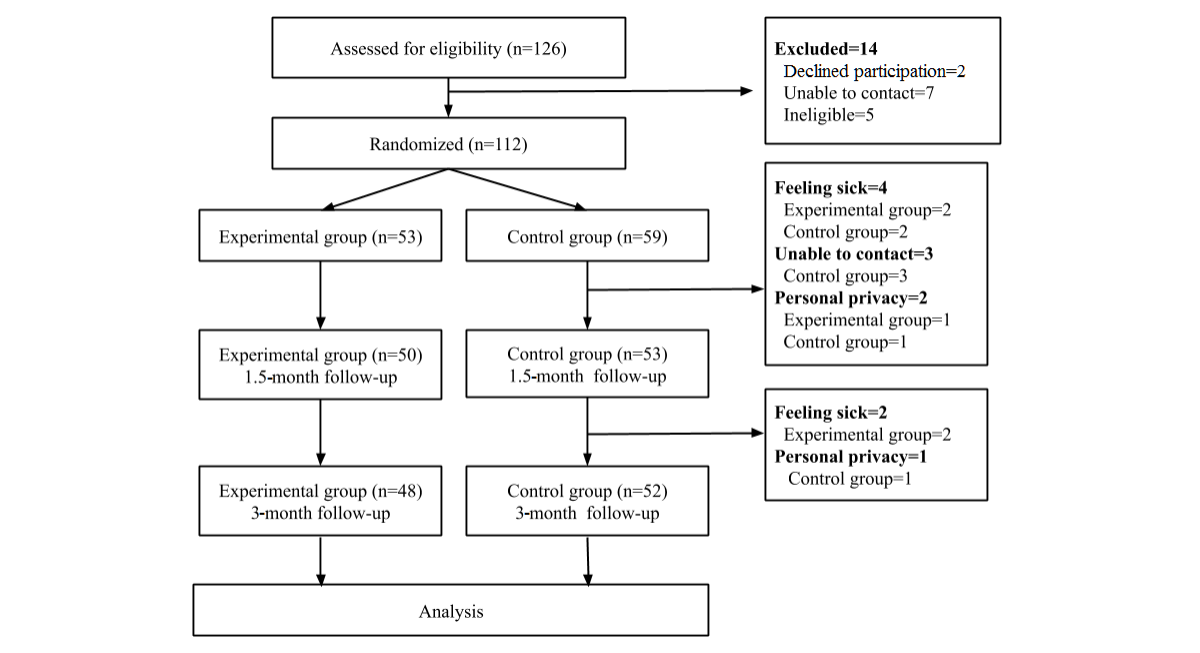
Study flowchart.

Randomization and attrition data were organized according to the Consolidated Standards Of Reporting Trials (CONSORT) guidelines [43] (see Figure 1). A total of 112 eligible women, after initial diagnosis of nonmetastatic breast cancer, were enrolled in the study and randomly allocated to the experimental (n=53) or control (n=59) group. Reasons for patient dropout included feeling sick during treatment (n=6), lost to follow-up (n=3), and concern for their personal privacy (n=3). A total of 48 participants remained in the experimental group and 52 remained in the control group at the 3-month follow-up; the follow-up completion rate was 89.3% (100/112). The statistical power of 100 participants was 0.99, which is adequate according to Cohen [44].

At baseline, all participants were assessed using paper-based instruments. At the 1.5-month follow-up evaluation, half of the participants were assessed using Web-based instruments: 48 used paper-based forms versus 55 who used Web-based forms. At the 3-month follow-up evaluation, most participants were assessed using Web-based instruments: 22 used paper-based forms versus 78 who used Web-based forms. The internal consistency reliability of the two groups at each follow-up were adequate; the Cronbach alpha levels are shown in the Instruments section.

### Participant Demographics

The demographics and baseline QoL from the two groups were similar. The largest age group was 50-64 years (51/112, 45.5%). Participants had bachelor’s degrees or equivalent levels of education (34/112, 30.4%). Over half (78/112, 69.6%) of the participants were married. One-third of participants had two children (43/112, 38.4%). Close to half (52/112, 46.4%) of the participants were employed. The majority of participants did not have any comorbid diagnoses (98/112, 87.5%). The largest number of participants had stage II breast cancer (44/112, 39.3%) and received breast conservation surgery (76/112, 67.9%), chemotherapy (73/112, 65.2%), radiotherapy (74/112, 66.1%), and hormone therapy (60/112, 53.6%). There were no statistical differences between the control and experimental group participants with regard to any of the baseline characteristics. Detailed statistical results are shown in Table 2.

At the 1.5-month and 3-month follow-up evaluations, there were also no statistical differences between control and experimental group participants with respect to receiving chemotherapy (at 1.5 months, *P*=.40; at 3 months, *P*=.76), radiotherapy (at 1.5 months, *P*=.92; at 3 months, *P*=.31), and hormone therapy (at 1.5 months, *P*=.13; at 3 months, *P*=.06), which also showed homogeneity in the participants’ medical treatment statuses at the two follow-up evaluations.

**Table 2:** Demographic characteristics and treatments of the participants.

Characteristic and treatment		Experimental group (n=53), n (%)		Control group (n=59), n (%)		All participants (N=112), n (%)		*P* value	
**Age in years**								.33	
	20-34		8 (15)		4 (7)		12 (10.7)		
	35-49		22 (42)		27 (46)		49 (43.8)		
	50-64		23 (43)		28 (47)		51 (45.5)		
**Education**								.05	
	Junior high school		4 (8)		12 (20)		16 (14.3)		
	Senior high school		14 (26)		14 (24)		28 (25.0)		
	College		9 (17)		13 (22)		22 (19.6)		
	Bachelor or equivalent		18 (34)		16 (27)		34 (30.4)		
	Master or equivalent		8 (15)		4 (7)		12 (10.7)		
**Marital status**								.21	
	Unmarried		13 (25)		10 (17)		23 (20.5)		
	Married		37 (70)		41 (69)		78 (69.6)		
	Divorced		1 (2)		6 (10)		7 (6.3)		
	Widowed		2 (4)		2 (3)		4 (3.6)		
**Number of children**								.28	
	0		16 (30)		15 (25)		31 (27.7)		
	1		7 (13)		7 (12)		14 (12.5)		
	2		22 (42)		21 (36)		43 (38.4)		
	More than 2		8 (15)		16 (27)		24 (21.4)		
**Work status**								.79	
	Unemployed		6 (11)		8 (14)		14 (12.5)		
	Housewife		18 (34)		20 (34)		38 (33.9)		
	Retired		4 (8)		4 (7)		8 (7.1)		
	Employed		25 (47)		27 (46)		52 (46.4)		
**Past disease history**								.18	
	No		44 (83)		54 (92)		98 (87.5)		
	Yes		9 (17)		5 (8)		14 (12.5)		
**Breast cancer stage**								.55	
	0		5 (9)		4 (7)		9 (8.0)		
	I		22 (42)		20 (34)		42 (37.5)		
	II		17 (32)		27 (46)		44 (39.3)		
	III		9 (17)		8 (14)		17 (15.2)		
**Surgery**								.55	
	Conservation therapy		38 (72)		38 (64)		76 (67.9)		
	Mastectomy		15 (28)		21 (36)		36 (32.1)		
**Chemotherapy**								.32	
	No		21 (40)		18 (31)		39 (34.8)		
	Yes		32 (60)		41 (69)		73 (65.2)		
**Radiotherapy**								.11	
	No		14 (26)		24 (41)		38 (33.9)		
	Yes		39 (74)		35 (59)		74 (66.1)		
**Hormone therapy**								.08	
	No		20 (38)		32 (54)		52 (46.4)		
	Yes		33 (62)		27 (46)		60 (53.6)		

### Changes in Quality-of-Life Questionnaire Core 30 Indicators

Before instruction (T0), the mean total summary scores of the QLQ-C30 for the experimental and control groups were 74.47 (SD 14.96) and 78.30 (SD 12.59), respectively, with no difference between the groups (*P*=.14).

At the 1.5-month and 3-month follow-up evaluations, these scores were 79.13 (SD 15.31) and 83.45 (SD 10.85) in the experimental group, respectively, and 79.49 (SD 12.41) and 82.23 (SD 12.07) in the control group, respectively. The mean summary scores for the QLQ-C30 in both groups showed a significant improvement by 3 months: the difference between the T2 and T0 scores was 8.98 in the experimental group and 3.93 in the control group. GEE analysis showed a statistically significant difference (*P*=.03) in the interaction between groups and time, which meant the experimental group had a greater mean summary score for the QLQ-C30 (difference=5.05) than the control group after the intervention at the 3-month assessment (see Table 3).

**Table 3:** Generalized estimating equation analysis of longitudinal outcome of the Quality-of-Life Questionnaire Core 30 (N=112).

Step	Functional scales^a^, mean				*P* value		Symptom scales^b^, mean				*P* value		Global health status or quality of life^a^, mean				*P* value		Total summary scores^a^, mean				*P* value
	Exp^c^	Con^d^	Diff^e^			Exp		Con	Diff			Exp		Con	Diff			Exp		Con	Diff		
T0^f^	73.59	77.18	-3.58	.25		24.71		20.99	3.73	.16		57.08		63.28	-6.20	.08		74.47		78.30	-3.82	.14	
T1^g^	79.64	78.63	1.01	.71		21.14		19.50	1.64	.52		68.33		70.75	-2.42	.51		79.13		79.49	-0.37	.89	
T2^h^	82.76	80.64	2.12	.38		16.10		16.62	-0.52	.79		73.44		74.36	-0.92	.76		83.45		82.23	1.22	.55	
T1-T0	6.05	1.46	4.59	.06		-3.58		-1.49	-2.09	.32		11.26		7.48	3.78	.38		4.65		1.19	3.46	.09	
T2-T0	9.17	3.47	5.71	.04		-8.61		-4.37	-4.25	.06		16.36		11.08	5.28	.18		8.98		3.93	5.04	.03	

^a^Higher scores correspond with better quality of life.

^b^Lower scores correspond with better quality of life.

^c^Exp: experimental group.

^d^Con: control group.

^e^Diff: difference between the experimental and control groups.

^f^T0: baseline or pretest evaluation.

^g^T1: 1.5-month follow-up evaluation.

^h^T2: 3-month follow-up evaluation.

### Changes in Breast Cancer-Specific Quality-of-Life Questionnaire Indicators

Before instruction (T0), the QLQ-BR23 mean summary scores of the experimental and control groups were 59.68 (SD 12.46) and 61.21 (SD 12.65), respectively, with no difference between the groups (*P*=.52).

At 1.5-month and 3-month follow-up evaluations, these scores were 62.56 (SD 13.10) and 65.53 (SD 10.29) in the experimental group, respectively, and 62.13 (SD 12.74) and 63.13 (SD 12.66) in the control group, respectively. The mean summary scores for QLQ-BR23 in both groups increased significantly by 3 months: the difference between the T2 and T0 scores was 5.85 in the experimental group and 1.92 in the control group. GEE analysis showed a statistically significant difference (*P*=.04) in the interaction between groups and time, which meant the experimental group had a greater total summary score for the QLQ-BR23 (difference=3.93) than the control group after the intervention at the 3-month assessment (see Table 4).

**Table 4:** Generalized estimating equation analysis of longitudinal outcome of the Breast Cancer-Specific Quality-of-Life Questionnaire (N=112).

Step	Functional scales^a^, mean			*P* value	Symptom scales^b^, mean			*P* value	Total summary scores^a^, mean			*P* value
	Exp^c^	Con^d^	Diff^e^		Exp	Con	Diff		Exp	Con	Diff	
T0^f^	46.54	47.32	-0.78	.76	27.38	25.45	1.93	.51	59.68	61.20	-1.53	.52
T1^g^	49.39	47.06	2.32	.35	25.11	24.08	1.03	.72	62.56	62.13	0.43	.86
T2^h^	34.65	32.82	1.83	.22	21.69	23.46	-1.78	.51	65.53	63.13	2.40	.24
T1-T0	2.85	-0.25	3.10	.17	-2.27	-1.37	-0.90	.71	2.88	0.93	1.95	.30
T2-T0	-11.89	-14.49	2.61	.31	-5.69	-1.99	-3.70	.14	5.85	1.92	3.93	.04

^a^Higher scores correspond with better quality of life.

^b^Lower scores correspond with better quality of life.

^c^Exp: experimental group.

^d^Con: control group.

^e^Diff: difference between the experimental and control groups.

^f^T0: baseline or pretest evaluation.

^g^T1: 1.5-month follow-up evaluation.

^h^T2: 3-month follow-up evaluation.

### Sensitivity Analysis

We performed a sensitivity analysis for the attrition cases using GEE analysis. The results showed that the mean summary scores also increased in the QLQ-C30 (difference between experimental and control groups was 5.05, *P*=.07) and the QLQ-BR23 (difference between experimental and control groups was 3.93, *P*=.07), with no statistically significant difference between the two groups noted at the 3-month assessment (see Table 5).

**Table 5:** Generalized estimating equation analysis of longitudinal outcome for the Quality-of-Life Questionnaire Core 30 (QLQ-C30) and the Breast Cancer-Specific Quality-of-Life Questionnaire (QLQ-BR23) for sensitivity analysis.

Step	QLQ-C30 mean summary scores			*P* value	QLQ-BR23 mean summary scores				*P* value
	Exp^a^	Con^b^	Diff^c^		Exp	Con	Diff		
T0^d^	74.47	78.30	-3.83	.14	59.68	61.21	-1.53	.52	
T1^e^	79.27	79.49	-0.22	.89	63.04	62.13	0.91	.87	
T2^f^	83.45	82.23	1.22	.99	65.53	63.13	2.40	.40	
T1-T0	4.80	1.19	3.61	.11	3.36	0.92	2.44	.34	
T2-T0	8.98	3.93	5.05	.07	5.85	1.92	3.93	.07	

^a^Exp: experimental group.

^b^Con: control group.

^c^Diff: difference between the experimental and control groups.

^d^T0: baseline or pretest evaluation.

^e^T1: 1.5-month follow-up evaluation.

^f^T2: 3-month follow-up evaluation.

## Discussion

### Principal Findings

Our team developed the BCSMS app with eight main features, in line with the results from our previous study, to support self-management for women after a first diagnosis of breast cancer. After 3 months of repeated QoL evaluation, the principal results showed that for the QLQ-C30, both groups had improvement in their functional scales (9.17 for the experimental group vs 3.47 for the control group), symptom scales (-8.61 for the experimental group vs -4.37 for the control group), global health status (16.36 for the experimental group vs 11.08 for the control group), and total summary scores (8.98 for the experimental group vs 3.93 for the control group). The BCSMS app user group participants had significant improvement in their functional scores (difference=5.71, *P*=.04) and total summary scores (difference=5.04, *P*=.03) compared to the control group.

Based on the results of the QLQ-BR23, neither group had improvement in functional scales (-11.98 for the experimental group vs -14.49 for the control group) by the third month. However, improvement was noted in symptom scales (-5.69 for the experimental group vs -1.99 for the control group) and total summary scores (5.85 for the experimental group vs 1.92 for the control group) by the third month. The BCSMS app user group had a significant improvement in total summary scores (difference=3.93, *P*=.04) when compared to the control group.

After sensitivity analysis (ie, no imputation for attrition cases), the total summary scores for the QLQ-C30 (difference=5.05, *P*=.07) and the QLQ BR23 (difference=3.93, *P*=.07) also showed improvement by the third month between the two groups but it was not significant. Based on these findings, further evaluation of QoL among women with a first diagnosis of breast cancer after using the BCSMS app is warranted.

### Comparison With Prior Work

The overall QLQ-C30 and QLQ-BR23 scores (see Tables 3 and 4) in both groups in this study were close to those from a multicenter, cross-sectional study in Taiwan [19]. They were also similar to the QoL scores for women after 1 year following a breast cancer diagnosis in a 10-year, long-term follow-up study in Germany [45]. This may be due to standardized medical treatments for each stage of breast cancer that were adopted regionally and globally [3-5]. Mobile health apps can effectively support patients with chronic disease self-management [46-48]. Patients with a first diagnosis of breast cancer need knowledge about the disease and to learn about self-management in order to live well with the cancer (eg, surgery-wound pain relief, lymphedema prevention, and controlling body weight). Others have demonstrated that mobile health apps are associated with improved knowledge of the disease and self-management, including self-efficacy with performing shoulder exercises during and after treatment, symptom relief, and QoL during chemotherapy [49-51]. In our study, the BCSMS app also demonstrated improved QoL for Taiwanese women with breast cancer. Among the eight main features included in the BCSMS app, which was developed with the user-centered approach [52], was a feature that provided evidence or *knowledge* about breast cancer (eg, cancer stages, treatments, side effects, lymphedema prevention, and relapse) and addressed basic information for the patients. According to the prior study, higher levels of anxiety were experienced by preoperative breast cancer patients who received information delivered via a mobile app than by patients who did not use the app [53]. In our study, every subject was postoperative; we did not know the BCSMS app would increase patients’ anxiety levels when they had *knowledge* about breast cancer before operation. Our results showed that the group who used the BCSMS app had higher scores in functional scales—physical functioning, role functioning, emotional functioning, cognitive functioning, and social functioning—than did the group who did not use the app at the 3-month follow-up. This might be because the BCSMS app not only provided the evidence or *knowledge* about breast cancer but also provided *emotional support* features to prevent anxiety and depression, including information on mental support, music therapy, mindfulness activities, sleeping well, and acupuncture point massage; *social resource* information, including information on nonprofit supporting organizations, financial support, and housekeeping; *experience sharing*, including encouraging information from other senior survivors and health care professionals; and *expert consulting*, including over 100 frequently asked questions. With this positive and useful information from the BCSMS app, patients were supported in overcoming cancer-related emotional disturbances.

In addition, the BCSMS app provided videos that demonstrated effective body movements to prevent lymphedema and low-intensity exercises in the *exercise and rehabilitation* feature. This feature may have reduced patients’ symptom scale scores, including for fatigue, pain, dyspnea, insomnia, appetite loss, and constipation. Lowering of these scores benefitted symptom control during the first year of cancer treatment (eg, cancer-related fatigue improvement) among experimental subjects and had positive outcomes, similar to those seen in prior studies [54,55].

In the *personal health record* feature for tracking treatments and side effects, subjects in the experimental group were able to record details about their personal medical treatments (eg, date of surgery, period of radiotherapy, medication for chemotherapy, and hormone therapy), record physical-related self-measurements (eg, body temperature and arm circumference), view data graphs, and receive abnormal-data warnings (eg, reminders to patients to consult their psychologists when their emotions were self-assessed as poor through the Brief Symptom Rating Scale) (publication is forthcoming). This feature was also similar to the patient-reported outcomes for symptom monitoring during routine cancer treatment; the results showed that integration of patient-reported outcomes into the routine care of patients with metastatic cancer was associated with increased survival compared with usual care [56]. Having all of the features integrated into one mobile app, as well as the app’s accessibility and ease of use regarding breast cancer self-management, may have contributed to the improvement in the patients’ QoL.

### Limitations

There were two main limitations of this study. The first was maturation bias, as our study followed participants for only 3 months. Although the control group did not use the BCSMS app, they still had supportive care from health care professionals (eg, physicians and oncology case managers), and perhaps their information-searching competence helped them with self-management and to increase their QoL. The second was that we did not know the actual frequency of use of the BCSMS app among the experimental group. To prevent the Hawthorne effect (ie, causing nonroutine behavior as a result of being observed) [57], the research team did not remind the subjects in the experimental group to use BCSMS app. Such limitations might have influenced the results in this study.

### Conclusions

The purpose of the study was to investigate the QoL of women with breast cancer in Taiwan after using the BCSMS app. According to the results, women with a first diagnosis of breast cancer appear to have experienced increased QoL after receiving cancer treatment. The BCSMS app provided users with supportive evidence to promote their QoL, compared with those who did not use the app. This might be because the BCSMS app delivered comprehensive information (eg, eight main features developed during our prior work) to women with breast cancer when they were first diagnosed and the app and its features supported their needs during their treatment. Next steps would be to introduce the BCSMS app to more breast cancer patients and to further evaluate whether QoL is sustained among these patients.

### Implications

Using objective data, such as users’ log-in frequencies, most-used features, and content analysis from the *personal health record* for treatment and side effects, could be investigated to better understand how the BCSMS app helps women with breast cancer. In addition, subjective feedback from BCSMS app users is important for future modifications and enhancements of the app. In the future, the BCSMS app could be introduced to breast cancer patients in different countries after culturally sensitive content is added (eg, *diet and nutrition* for breast cancer patients and *social resource* information) and when multiple languages can be incorporated into the interface (eg, English).

## Appendix

Multimedia Appendix 1 CONSORT-EHEALTH checklist (V 1.6.1).

## Abbreviations

BCSMS: breast cancer self-management support
CONSORT: Consolidated Standards Of Reporting Trials
EORTC: European Organization for Research and Treatment of Cancer
GEE: generalized estimating equation
IRB: Institutional Review Board
MAR: missing at random
QLQ: Quality-of-Life Questionnaire
QLQ-BR23: Breast Cancer-Specific Quality-of-Life Questionnaire
QLQ-C30: Quality-of-Life Questionnaire Core 30
QoL: quality of life
T0: pretest evaluation
T1: 1.5-month follow-up evaluation
T2: 3-month follow-up evaluation
